# Supplementary material for: Spatial distribution of common childhood illnesses, healthcare utilisation and associated factors in Ethiopia: Evidence from 2016 Ethiopian Demographic and Health Survey
Source: PLoS One. 2023 Mar 10;18(3):e0281606. doi: 10.1371/journal.pone.0281606 (PMC10004611; doi:10.1371/journal.pone.0281606)
Supplement: Attachment — Submitted filename: Reviewers comment.docx [file pone.0281606.s001.docx]

- There is a 2020 EDHS report, authors should use more recent data for analysis as changes might of occurred in the country that might have improved or increased prevalence of childhood diseases and utilization of services
- The section on study design was used to describe Ethiopia instead of telling us the type of study, the target population. The study would benefit from a revision of this section.
- What do the authors mean by “probability proportional to segment size”.
- “A total of 10,417 caregivers or mothers of under-five children were interviewed” contradicts the sample size information in the abstract and the result section “10,641**”**
- “Sick” or “Seek”. I observed the use of the word sick a couple of times. Please correct the word as appropriate
- The term “the two-week prevalence of common childhood illnesses “is not clear. Am not sure this is the term used in the DHS report. Authors need to ensure consistency of terms compared to the EDHS report.
- “Table 1 shows the outcomes and potential predictors for health service utilization”. Table 1 didn’t differentiate between outcomes and predictors. Update table 1 to contain all these variables “age and sex of the child, woman-headed household, age of the mother or caregiver, mothers’ or caregivers’ education level, household size, wealth index, and distance to health facility”.
- This statement “The 94 clusters without sick children and 18 clusters from Somali region without shape files were excluded in the spatial analysis. Therefore, the results of the spatial analysis are based on 531 clusters” however it should be a part of methodology as a limitation of the study
- In the result section, I had expected to see the prevalence of each of the childhood disease which were mentioned in the methodology section however am seeing a description of the prevalence. Authors should endeavor to present the prevalence of the childhood disease
- “*lack adequate number of primary sampling units or observation per stratum to estimate the standard error to get the 95% CI for acute respiratory infection and diarrhoea”. Are you saying only one primary sampling unit had people seeking health care? I think authors should endeavor to check this analysis to ensure accuracy.
- Table 4 seems to be a description of measures. I believe this should be a part of the methodology section
- The figure 1 maps aren’t correct. Prevalence is percentage vales and should represent an area. Dot distribution analysis can’t be used to represent a prevalence analysis. Authors should replot the maps
- Figure 2 don’t represent spatial patterns. Please change the distribution curves to maps. This analysis is not properly carried out
